# Low Levels of Omega-3 Long-Chain Polyunsaturated Fatty Acids Are Associated with Bone Metastasis Formation in Premenopausal Women with Breast Cancer: A Retrospective Study

**DOI:** 10.3390/nu12123832

**Published:** 2020-12-15

**Authors:** Caroline Goupille, Philippe G. Frank, Flavie Arbion, Marie-Lise Jourdan, Cyrille Guimaraes, Michelle Pinault, Gilles Body, Stephan Chevalier, Philippe Bougnoux, Lobna Ouldamer

**Affiliations:** 1 Department of Gynecology, Centre Hospitalier Régional Universitaire de Tours, Hôpital Bretonneau, 2 Boulevard Tonnellé, 37044 Tours, France; body@univ-tours.fr (G.B.); l.ouldamer@chu-tours.fr (L.O.); 2 Laboratoire N2C « Nutrition, Croissance et Cancer », Université de Tours, INSERM UMR1069, 10 Boulevard Tonnellé, 37032 Tours, France; philippe.frank@univtours.fr (P.G.F.); ml_jourdan@yahoo.fr (M.-L.J.); guimaraes-carneiro@univ-tours.fr (C.G.); mpinault@univ-tours.fr (M.P.); stephane.chevalier@univ-tours.fr (S.C.); philippe.bougnoux@univ-tours.fr (P.B.); 3 Department of Pathology, Centre Hospitalier Régional Universitaire de Tours, Hôpital Bretonneau, 2 Boulevard Tonnellé, 37044 Tours, France; f.arbion@chu-tours.fr

**Keywords:** breast cancer, bone metastases, *n-*3 long-chain polyunsaturated fatty acids, adipose tissue

## Abstract

In the present study, we investigated various biochemical, clinical, and histological factors associated with bone metastases in a large cohort of pre- and postmenopausal women with breast cancer. Two hundred and sixty-one consecutive women with breast cancer were included in this study. Breast adipose tissue specimens were collected during surgery. After having established the fatty acid profile of breast adipose tissue by gas chromatography, we determined whether there were differences associated with the occurrence of bone metastases in these patients. Regarding the clinical and histological criteria, a majority of the patients with bone metastases (around 70%) had tumors with a luminal phenotype and 59% of them showed axillary lymph node involvement. Moreover, we found a negative association between the levels of *n-*3 long-chain polyunsaturated fatty acids (LC-PUFA) in breast adipose tissue and the development of bone metastases in premenopausal women. No significant association was observed in postmenopausal women. In addition to a luminal phenotype and axillary lymph node involvement, low levels of *n-*3 LC-PUFA in breast adipose tissue may constitute a risk factor that contributes to breast cancer bone metastases formation in premenopausal women.

## 1. Introduction

Breast cancer is the most commonly diagnosed cancer in women and is the leading cause of cancer death with 2,088,849 new cases and 626,679 specific mortality worldwide in 2018 [1]. For this cancer, bone is the most common site of metastasis, as it is present in about 70% of all metastatic breast cancer patients regardless of the menopausal status [2]. Bone metastases (BM) carry out a fairly good prognosis compared to other metastatic sites. Nevertheless, BM are associated with an important risk of developing skeletal-related events that negatively impact quality of life, mobility, and patient survival.

Several clinical studies have been conducted to identify the demographic, clinical, pathological, and genetic factors influencing BM development in breast cancer patients. They have reported a number of risk factors for BM, and they include axillary lymph node involvement, a large tumor size, and expression of the estrogen receptor by the tumor. Tumor phenotype is, therefore, widely accepted as a major risk factor since patients with luminal subtype tumors are at an increased risk of developing BM compared to patients with other breast cancer phenotype [2]. In a retrospective study of 9625 women, researchers investigated different models to define the clinical risk factors that are associated with bone-only metastasis. Age and tumor phenotype (luminal) were reported to be the most relevant risk factors [3]. Formation of clinically evident bone metastases represents the final step of a complex process of interactions between tumor cells and the bone environment. However, tumor cells appear to be able to remain dormant for decades in patients, who experience disease relapse years after surgical excision of their primary tumor. A meta-analysis of over 4700 bone marrow aspirates from breast cancer patients without bone metastases showed that premenopausal patients have a significantly more elevated prevalence of bone marrow tumor cell dissemination than postmenopausal women (32.7% versus 29.5%). These data suggest that the premenopausal bone marrow can promote the attraction and/or retention of tumor cells [4].

Bisphosphonate remains the drug of choice to limit bone resorption, pain, and skeletal-related events, in addition to delay bone metastasis recurrence. Nonetheless, a collaborative meta-analyses from the early Breast Cancer Trialists’ Collaborative Group has recently observed reduced bisphosphonate or other drug efficacy in the treatment of bone lesions in premenopausal patients compared to postmenopausal patients [5]. This observation may suggest that the mechanism of action of these drugs in the bone microenvironment may be hormone dependent [6].

The molecular pathways that regulate bone metastasis formation involve several steps, which include breast cancer cell detachment from the primary site, homing to the bone site with attachment and extravasation through the vascular barrier, colonization, and proliferation of breast cancer cells in the bone with a complex crosstalk between breast cancer cells and the bone microenvironment [7,8,9]. In 1889, Paget and his colleagues were the first to propose the seed-and-soil hypothesis, which proposes that circulating tumor cells can travel around the whole body but a few survive and form metastatic lesions [10]. Although the seed-and-soil theory is still used to explain the phenomenon of orga*n-*specific metastases, several studies have proposed alternative or complementary hypotheses to explain the existence of preferred metastasis sites for each cancer type or determine how metastasis growth can be supported by the host tissue microenvironment [11]. Similar to osteoporosis, breast cancer BM are of the osteolytic type, and they are related to osteoclast activation supported by inflammatory factors. Osteoporosis has not been associated with an increased risk of bone metastasis formation but a weakened bone microenvironment can accelerate bone metastasis progression [12].

The lipid composition of the diet in terms of saturated, monounsaturated, and *n-*6 and *n-*3 polyunsaturated fatty acids (SFA, MUFA and *n-*6 PUFA and *n-*3 PUFA, respectively) content may be associated with a modulation of breast cancer risk [13]. In addition, a diet enriched in *n-*3 polyunsaturated fatty acids (*n-*3 PUFA) has been shown to have beneficial effects in various types of cancer including breast cancer [14,15]. The fatty acid composition of a diet (e.g., SFA, MUFA, and *n-*6 and *n-*3 PUFA) can also be evaluated by food frequency questionnaire. Nevertheless, numerous studies have shown that the fatty acid composition of adipose tissue can directly be used as a surrogate for the evaluation of past lipid intake [16]. In that case, the use of adipose tissue lipid content as a biomarker could circumvent the known bias associated with the food frequency questionnaire queries. Having low fatty acid turnover [17,18], the lipid composition of adipose tissue is considered as a biomarker of choice of long-term dietary intake of fatty acids [16,19]. This association between diet and adipose tissue lipid composition has also been observed in breast adipose tissue when women are supplemented with *n-*3 long-chain polyunsaturated fatty acids (LC-PUFA) capsules [20].

Several preclinical studies have reported that *n-*3 LC-PUFA-enriched diets can reduce breast tumor and bone metastasis formation [21,22], but no study in human has reported a potential association between fatty acids that include *n-*3 LC-PUFA and bone metastasis. In the present study, we selected more than 250 breast cancer patients and examined if the levels of fatty acids (SFA, MUFA, and PUFA) stored in breast adipose tissue are associated with the onset of bone metastasis formation according to the menopausal status. For this purpose, clinical and histological parameters were collected and the fatty acid profile of the mammary adipose tissue was determined by biochemical analysis (via gas chromatography) of cryopreserved adipose tissue samples.

## 2. Materials and Methods

### 2.1. Study Population

In this retrospective study, we analyzed data from consecutive women treated for invasive breast cancer between January 2009 and June 2012 at our Tertiary Breast Care center in Tours (France). The population of patients was from Central France with mostly Caucasian origins. The present study was exploratory in nature, and we initially planned to include approximately 250 consecutive patients with tumors representative of all breast cancer phenotypes and for whom adipose tissue biopsies had previously been harvested for fatty acid analysis of triglyceride. The inclusion criterion was women treated between January 2009 and June 2012 for invasive breast cancer for which the associated breast adipose tissue samples had been excised during surgery and stored in our University Hospital cryobank. To have a substantial number of patients with the less frequent HER2 phenotype (*n* = 40 HER2), all patients of this group with tissues harvested and stored in the cryobank between January 2009 and June 2012 were selected using the cryobank database. We also selected all the adipose tissues associated with triple negative tumors (*n* = 67 triple negative) from the same period. As expected, during this period, the majority of breast tumor phenotypes were of the luminal phenotype, and a selection had to be made. Patients with luminal A or B tumor phenotypes (92 samples for luminal A and 87 for luminal B) were also selected throughout the same period according to sample availability and alphabetical order, and they were chosen to match the age distribution of the HER2 and triple negative tumor-carrying patient groups. As much as it was possible, samples of the luminal phenotype groups were matched with HER2 and triple negative patients of the same year. To prevent a potential selection bias, staff involved in patient selection (L. Ouldamer and C. Goupille) was only aware of the patient first letters’ name and their date of birth as well as the corresponding tumor phenotype. A total of 286 samples were selected at the beginning for the study. However, 16 samples were not found in the cryobank, and 9 were not large enough to perform a complete lipid analysis. Therefore, we used 261 tissues samples for which we were able to obtain a complete fatty acid profile. The main outcome was fatty acid composition of mammary adipose tissue according to the occurrence or not of bone metastasis in women managed for invasive breast cancer. The secondary outcome fatty acid composition of mammary adipose tissue according to the occurrence or not of bone metastasis stratified on patient’s menopausal status.

Histological and immunohistochemistry (IHC) analyses were performed by an experienced pathologist (F. Arbion). This pathologist reviewed histology reports, biopsy slides, surgical excision specimens, and determined grade, and tumor phenotype using the estrogen/progesterone receptors and HER2 expression profile (Agilent, Les Ulis, France, Cat# M7047, RRID:AB_2101946 for estrogen receptor, Cat# M3569, RRID:AB_2532076 for progesterone receptor, Cat# A0485, RRID:AB_2335701 for HER-2).

The adipose tissue collection cryobank was authorized and declared to the French Ministry of higher Education and Research (reference number DC-2008-308), and the study was performed with approval of the Tours University Teaching Hospital Review Board.

Clinical information was obtained using medical records held at our institution in a computerized database. Post-treatment imaging consisted of breast/chest ultrasound/mammograms once a year. Follow-up visits were conducted every 4 months for the first 2 years, every 6 months for the following 3 years, and once a year thereafter. Disease recurrence was diagnosed by biopsy or imaging techniques adapted to clinical symptoms (computed tomography and/or magnetic resonance imaging, or 18-fluorodeoxy glucose positron emission tomography (18-FDG PET CT). Time-to-event analyses were calculated from the date of primary surgery as the starting point and women who were alive and without recurrence were censored at the date of last follow-up. During follow-up, the occurrence of BM was recorded and presented according to the menopausal status.

### 2.2. Samples Collection

Breast adipose tissue samples and lipid analysis were obtained from 261 patients. Samples were excised during surgery from the external (tumor-free) region of the lumpectomy or mastectomy, and they were stored in liquid nitrogen to avoid degradation until lipid analysis.

### 2.3. Lipid Analysis

Determination of the adipose tissue fatty acid content was performed as previously described [19,20]. Briefly, lipids were extracted by the Folch method [21], and triglycerides were purified by preparative thin layer chromatography (cat# 1057150001, Millipore, Guyancourt, France). Fatty acids were methylated using boron trifluoride (CAS Number: 2802-68-8) in a methanol solution (Sigma-Aldrich, Saint-Quenti*n-*Fallavier, France) and analyzed by capillary gas chromatography (GC-2010 Plus chromatograph, Shimadzu, France) using a polar column (BPX70 column, 60 m × id 0.25 mm, cat#054623, SGE analytical Science, Courtaboeuf, France) and an o*n-*column injector. Chromatography analysis was supervised and validated by two experienced analytical biochemists (C. Goupille and M. Pinault) blinded to the clinical data. Fatty acid levels were expressed as percentages of the total integrated area. In this study, long-chain fatty acids included fatty acids containing between 20 and 24 carbons.

### 2.4. Statistics

Characteristics of women, tumor, and adipose tissue composition were analyzed using Chi-square statistics or Fisher’s exact test for categorical variables and *t*-test or analysis of variance (ANOVA) for continuous variables. The main outcome measure in the present study was the occurrence of BM. A *p*-value of less than 0.05 was considered statistically significant. Data were managed with an Excel database (Microsoft, Redmond, WA, USA) and analyzed with the R 3.0.2 software available online (R Project, Free Software Foundation’s GNU project, Lucent Technologies, Murray Hill, NJ, USA).

## 3. Results

Characteristics of the population are described in Table 1. Among the 261 women, 53 (20.3%) developed bone metastasis (BM) during the follow-up (median 62 months). Between both groups with or without BM, age, BMI, or phenotypes were not significantly different. However, patients with bone metastasis presented reduced tumor grade (*p* = 0.01) and the proportion of patients with positive axillary lymph node was significantly increased (54.7%) compared to patients without bone metastasis (39.5%) (*p* = 0.01).

Among the included women, 159 of them were postmenopausal, among which 32 (20.1%) presented bone metastasis, and 99 presented a premenopausal status, among which 21 (21.2%) presented BM. Three patients (1%) had an unknown menopausal status.

The main outcome was to explore fatty acid composition of mammary adipose tissue according to the occurrence or not of bone metastasis in women managed for invasive breast cancer. No significant differences were observed in terms of fatty composition between patients with BM and patients without BM (data not shown).

The main fatty acids observed in breast adipose tissue of premenopausal women are presented in Table 2, according to the presence of BM. Significant differences were found in the fatty acid profile of adipose tissue from patients with BM compared to patients without BM. *n-*3 LC-PUFA levels were significantly decreased in the adipose environment of women with BM (*p* = 0.02). When examining fatty acids individually, docosapentaenoic acid (DPA) levels were significantly decreased (*p* = 0.03), and the decrease in docosahexaenoic acid (DHA) levels was marginally significant (*p* = 0.06).

In postmenopausal women, levels of the main breast adipose tissue fatty acids are presented in Table 3, according to the presence of BM. No significant association with the development of BM was observed.

Finally, we compared the characteristics of patients with BM (Table 4). The proportion of patients with a luminal phenotype was not significantly different between premenopausal (58%) and postmenopausal women (77%). Regarding other metastatic sites, a little more than 60% of patients presented visceral metastases and 30% of them had brain metastases. However, these proportions were not different between pre- and postmenopausal women. It is also important to note that tumors from premenopausal patients with BM were also significantly larger compared to those observed in postmenopausal women.

## 4. Discussion

The aim of this study was to evaluate if alterations in breast adipose tissue lipid composition may be associated with the development of BM in women with breast cancer. A luminal phenotype and lymph node invasion are factors associated with BM, and we found a significant association between low levels of *n-*3 LC-PUFA in breast adipose tissue and the development of BM in premenopausal women.

Among the various clinical and histological factors examined, only lymph node involvement appears to contribute to BM, and a relationships between menopausal status or age and BM risk has not been clearly identified [2]. On the other hand, having a tumor with a luminal subtype is one of the most important risk factors associated with BM [2]. Our data are in agreement with this observation, which shows that patients with BM predominantly include pre- or postmenopausal patients with a luminal phenotype tumors and lymph node involvement.

Our results showed that low levels of *n-*3 LC-PUFA in breast adipose tissue are associated with the development of BM in premenopausal women. To explain the reduced levels of *n-*3 LC-PUFA in these patients, several hypotheses can be made. The first one is that PUFA depletion may be related to the presence of a tumor and its metabolism. To supply precursors for the synthesis of membrane phospholipids, fatty acids may be synthetized from nutrients (i.e., glucose or amino acids) or directly obtained from the microenvironment. In the past few years, reprogramming of fatty acid metabolism has been the focus of new studies in cancer cells, and targeting lipid metabolism in this context may become a new therapeutic approach for cancer treatment [23]. A few studies have pointed to the possibility of an elevated fatty acids—in particular PUFAs—consumption by the tumor. This observation was confirmed in colon cancer patients, who display reduced levels of circulating PUFAs [24] or in adipose tissues from patients with ovary cancer [25]. The study of Mika et al. [26] has shown for the first time an increased consumption of *n-*6 PUFAs and *n-*3 PUFAs in colonic cancer cells compared to normal colonocytes. Low levels of PUFA in adipose tissue may also be observed when specific types of PUFAs are released by adipose tissue during lipolysis [27]. To date, these observations have not been made in breast cancer. However, these studies have shown that levels of both types of PUFAs (i.e., *n-*6 PUFA and *n-*3 PUFA) were reduced [24,25,26], and it suggests that the metabolism in place is not necessarily targeting a specific type of LC-PUFAs. A consumption of *n-*3 LC-PUFAs by breast tumors suggests other hypotheses, i.e., the specific consumption of *n-*3 LC-PUFAs associated with the expression of specific fatty acid transporters (for transporters see review [28]). To date, the existence of a specific transport for *n-*3 or *n-*6 PUFAs has not been fully addressed [29], but the high affinity of FAPB7 (fatty acid binding protein 7) for DHA (docosahexaenoic acid) has previously been demonstrated [30]. If the hypothesis of a particular consumption of *n-*3 LC-PUFA by tumor cells is correct, these fatty acids are mainly used for the synthesis of ATP via beta oxidation. However, the incorporation of PUFAs into phospholipids can also alter the membrane physical properties and regulate downstream signaling pathways [31]. For example, it is important to note that *n-*3 LC-PUFAs tend to decrease the activation of signaling pathways associated with cellular proliferation [32]. In addition, PUFAs can directly act via their association to nuclear receptors such as PPARs, and they are capable of regulating lipid metabolism in cancer cells [33,34]. They may also generate active metabolites [35]. In that regard, metabolites derived from *n-*3PUFA appear to have opposite effects to those derived from *n-*6PUFA. With a deficiency in *n-*3 LC-PUFA, these regulatory mechanisms may not occur.

The second hypothesis to explain the low levels of *n-*3 LC-PUFA in adipose tissue of premenopausal women may be reduced *n-*3 LC-PUFA intake. In fact, the best suppliers of *n-*3 LC-PUFA (such as EPA, DPA, and DHA) are fish oil, marine fatty fishes, and seafood. *n-*3 LC-PUFA are essentially obtained via food intake since their endogenous synthesis (by elongation and desaturation) from the essential fatty acid alpha linolenic acid (ALA, 18: 3*n-*3) is extremely reduced in human (less than 0.2% of ALA is converted into DPA or DHA) [36].

Several studies have identified tumor cell profiles associated with bone metastasis formation [37,38,39], but the spread of breast cancer cells to bone and their survival in this new environment is not only influenced by the genetic signature of tumor cells, but multiple factors influencing the success or failure of the tumor cell evasion and its establishment at a distant tissue have been identified [40,41]. Host cells and soluble locally produced (paracrine) factors or from distant sites (endocrine) may influence metastasis development. In a mouse model, bone physiological changes were shown to increase breast cancer cell implantation in the bone, and osteoclast activity can promote the emergence of dormant cells scattered in the bone [42,43]. Several experimental studies have shown that *n-*3 LC-PUFA-enriched diets can reduce breast tumor bone metastasis formation [21,22]. Among the mechanisms involved, a modulation by *n-*3 LC-PUFA of the mammary tumor microenvironment [21], a reduction of M-CSF released by tumor cells [44], a decrease in CD44 (protein associated with epithelial mesenchymal transdifferentiation implicated in the metastatic potential) [22], or a modulation of the prosurvival/proliferative effects of estrogen [45] have been demonstrated. Rahman and colleagues concluded that *n-*3 LC-PUFA can attenuate breast cancer bone metastasis formation and the associated osteolysis by inhibiting the migration of breast cancer cells to the bone as well as by inhibiting osteoclastic bone resorption [46]. Several hypotheses can, therefore, be elaborated to explain how low levels of *n-*3 LC-PUFA may associated with bone metastasis. They include action on primary tumor cells and modification of their metastatic capabilities and regulation of tumor cell homing to the bone via CXCR4 [47]. *n-*3 LC-PUFA deficiencies may lead to foci of bone degradation, which provides a hospitable environment for metastasis formation.

Beneficial effects of *n-*3 LC-PUFA for bone health are well illustrated in the literature with experimental models. The skeletal metabolism is sensitive to *n-*3 LC-PUFA-deficient or -enriched diets [48,49]. Several mechanisms have been proposed, and they include the regulation of osteoclast and osteoblasts genesis via PPAR and COX/LOX metabolites [50]. More recently, data have shown that *n-*3 LC-PUFA supplementation can potentiate mesenchymal stem cell differentiation and increased osteogenesis [51], suppress human monocytes-derived osteoclasts formation via PPARs receptors [52,53], inhibit osteoclasts formation and bone resorption through GPR120 receptor [54], or inhibit RANK-L-induced osteoclasts formation [55]. In human, studies majoritarily showed that enriched *n-*3 LC-PUFA diets are associated with benefits for bone health, and these data have been essentially reported in postmenopausal women in an osteoporosis context [56]. Importantly, in one study exploring mineral bone density in patients with a vega*n-*like diet with an almost total absence of *n-*3 fatty acids, a decrease in bone mineral density was observed [57]. Taken together, these data highlight a downregulation of osteoclast and an upregulation of osteoblasts activity by *n-*3 LC-PUFA. Insufficient intake of *n-*3 PUFA may create a bone imbalanced microenvironment favorable to metastases implantation and/or development.

The final interrogation is why is the association between low levels of *n-*3 LC-PUFA and bone metastases only observable in premenopausal women. Estrogen is known to promote breast cancer cell proliferation. Importantly, fatty acid metabolism can interfere with estrogen activity and vice versa. A study examining mRNA levels in tumors has shown that tumors with luminal phenotypes favor endogenous fatty acid synthesis, whereas tumors that do not express hormonal receptors are more likely to import fatty acids [58]. Accordingly, the hormonal background may have a direct impact on lipid metabolism since the expression levels of several enzymes involved in fatty acid synthesis are diminished when the estrogen receptor is inactive [58]. It is also possible that reduced estrogen levels at menopause may induce similar effects. Moreover, studies have also shown that *n-*3 PUFA can alter estroge*n-*signaling pathways in the model luminal A MCF-7 cell line. In that case, it was shown that *n-*3 LC-PUFA (EPA and DHA) could inhibit estroge*n-*mediated proliferative signaling pathways and promote a proapoptotic pathway [45]. Taken together, these data suggest that beneficial *n-*3 LC-PUFA effects may be more observable in an estrogenic context in patients with hormone-sensitive tumors. Furthermore, taking into account diet consumption, studies have shown that adipose tissues (and blood) from postmenopausal women contain more *n-*3 LC-PUFA than those from premenopausal women [59]. The reasons for these differences have not been clearly identified, but a study has demonstrated the existence of alterations in DHA metabolism between young and older women [60]. We cannot exclude that this lipid metabolic change with menopause is responsible for a disruption in the association between low *n-*3LC-PUFA levels and bone metastasis formation during the postmenopause. In premenopausal women, double beneficial effects of *n-*3 LC PUFA on bone and cancer cells may explain, at least in part, the observed protective effects of *n-*3 PUFAs. The effects of DPA are less described in the literature, but its activities are consistent with those described for EPA and DHA [61].

Our study described for the first time in humans an association between *n-*3 LC PUFA levels and bone metastasis formation in premenopausal breast cancer patients. Although an efficient activity of bisphosphonates may be likely observed in postmenopausal women [5], *n-*3 LC-PUFA supplementation may be an interesting therapeutic approach to promote a beneficial remodeling of the bone microenvironment in premenopausal women. Two distinct bone microenvironments observed in pre- versus postmenopausal women have been reported, and different mechanisms in the development of bone metastasis according to the menopausal status have been described [62,63]. However, the impact of *n-*3 LC-PUFA on the bone microenvironment will have to be further explored in premenopausal women.

Some limitations deserve to be mentioned. First, we cannot exclude an inherent bias related to the retrospective and exploratory nature of the study, especially concerning the recurrence diagnosis, which represents a concern. However, all the included women were treated at our regional referral center applying the current French/European guidelines after systematic multidisciplinary committee approval. Second, we may not have recorded in full the data regarding subsequent sites of distant metastases after initial distant failure; although such an information may be more readily available in a retrospective institution review. We acknowledge that most events for luminal tumors may occur after many years of follow-up. Third, we did not have access to data regarding the eating habits of our patients. The reduced or lack of *n-*3 LC-PUFA dietary intake remains likely, but additional investigations will be required to support this hypothesis. Fourth, despite our precautions, we cannot exclude that the patient selection method, particularly that of patients with a luminal phenotype, may be biased. At the starting point, the objective of this project was to explore the potential relationships between breast cancer aggressiveness and fatty acid composition of breast adipose tissue. We had previously identified an association between inflammatory breast cancer and low levels of *n-*3 LC-PUFA [64]. In the present study, we report an association between bone metastases and low levels of adipose tissue *n-*3 LC-PUFA. Adipose tissue may serve as a biomarker of past lipid intake, but it appears that this statement is particularly more accurate for all PUFAs than SFA and MUFA, as previously and recently shown [16,65]. Additional studies characterizing the adipose tissue fatty acid profile, food frequency questionnaires, specific analyses of the tumor fatty acid metabolism, and the specific recruitment of patients with bone metastases will be required to identify the origin of the low levels of *n-*3 LC PUFA levels and eliminate a potential bias associated with the bone metastasis group size, which remains small in our study.

## 5. Conclusions

Alternative therapies are needed for premenopausal women targeting bone tumor cells and to maintain them in a dormant state or modify the bone microenvironment to make it less hospitable. While some experimental and epidemiological studies have suggested a beneficial effect of *n-*3 LC-PUFAs in cancer, international organizations recommend that current *n-*3 LC-PUFA intake be between 200 and 500 mg/day. Our retrospective and exploratory study shows that low levels of *n-*3 LC-PUFA in the adipose tissue of premenopausal women with breast cancer are associated with bone metastasis formation. Additional studies in human will be required to determine the origin of these low levels of *n-*3 LC-PUFA before proposing an *n-*3 LC-PUFA supplementation to prevent or contain bone metastasis in premenopausal women with breast cancer.

## Figures and Tables

**Table 1:** Demographic and histological characteristics of women with breast cancer.

	No Bone Metastasis *n* = 208		Bone Metastasis *n* = 53		Statistics
	Mean or *n* (%)	Range	Mean or *n* (%)	Range	*p*
Age (years)	57.0	28–89	55.9	33–84	0.49
Premenopausal	78 (37.5%)		21 (39.6%)		0.95
Postmenopausal	127 (61.1%)		32 (60.4%)		
UK menopausal status	3 (1.4%)		0		
HRT	33 (25.9%)		7 (21.9%)		1
BMI (Kg/m^2^)	25.2	16–40	25.5	13–41	0.78
Histological size (mm)	26.6	3–210	29.3	5–70	0.35
Molecular phenotype					0.45
Luminal A	67 (32.7%)		21 (39.6%)		
Luminal B	56 (27.3%)		16 (30.3%)		
Triple negative	51 (24.9%)		12 (22.6%)		
HER2	31 (15.1%)		4 (7.5%)		
Grade					0.01
-Grade 1	23 (11.0%)		0 (0%)		
-Grade 2	88 (42.3%)		30 (56.6%)		
-Grade 3	95 (45.7%)		21 (39.6%)		
-Unknown	2 (1%)		2 (3.8%)		
Lymphovascular invasion	58 (27.9%)		16 (30.2%)		0.53
Axillary positive LN	81 (38.9%)		29 (54.7%)		0.01
Multifocality	52 (25%)		8 (15.1%)		0.19

HRT: hormone replacement therapy; BMI: body mass index; LN: lymph node; HER2: Human epidermal growth factor receptor 2, UK: unknown.

**Table 2:** Gas chromatography assessment of the breast adipose tissue fatty acid composition according to the presence of bone metastasis in premenopausal women.

Fatty Acids *		No Bone Metastasis *n* = 78		Bone Metastasis *n* = 21		Statistics
		Mean	Range	Mean	Range	*p*
**Saturated**						
Myristic acid	14:0	3.47	1.65–4.90	3.56	2.47–4.72	0.14
Palmitic acid	16:0	23.61	19.63–28.8	23.9	21.01–26.63	0.52
Stearic acid	18:0	6.02	3.20–8.33	5.96	3.03–8.57	0.86
	Total SFA	34.11	25.97–40.62	34.37	29.85–40.12	0.68
**Monounsaturated**						
Myristoleic acid	14:1	0.27	0.01–0.56	0.30	0.14–0.60	0.38
Palmitoleic acid	16:1	3.34	1.09–8.45	3.56	1.80–6.89	0.54
Oleic acid	18:1*n-*9c	43.23	38.01–47.65	43.17	38.13–48.11	0.91
Vaccenic acid	18:1*n-*7c	1.79	1.25–2.86	1.81	1.39–2.56	0.81
	Total MUFA	49.94	42.48–56.19	50.15	45.92–55.71	0.77
**Polyunsaturated**						
Linoleic acid	18:2*n-*6c	10.63	6.43–21.0	10.37	7.39–15.05	0.59
Gamma linolenic acid	18:3*n-*6	0.04	0.02–0.09	0.04	0.02–0.06	0.31
Arachidonic acid	20:4*n-*6	0.29	0.15–0.63	0.30	0.16–0.60	0.92
	LC PUFA *n-*6	0.91	0.45–2.06	0.88	0.50-1.55	0.71
	Total *n-*6	11.61	6.94–22.68	11.33	8.30–16.22	0.57
Alpha linolenic acid	18:3*n-*3	0.60	0.29–1.04	0.58	0.37–0.98	0.62
Eicosapentaenoic acid	20:5*n-*3	0.07	0.02–0.16	0.06	0.03–0.11	0.15
Docosapentaenoic acid	22:5*n-*3	0.19	0.05–0.51	0.16	0.07–0.24	0.03
Docosahexaenoic acid	22:6*n-*3	0.16	0.03–0.35	0.13	0.05–0.21	0.06
	LC PUFA *n-*3	0.42	0.12–0.97	0.36	0.16–0.49	0.02
	Total *n-*3	1.05	0.63–1.52	0.97	0.60–1.43	0.11
***n-*6/*n-*3 ratio**	*n-*6/*n-*3	11.41	7.26–26.91	11.98	7.69–20.07	0.44

* expressed as % area, SFA: saturated fatty acids, MUFA: monounsaturated fatty acids, PUFA: polyunsaturated fatty acids, LC PUFA: long-chain polyunsaturated fatty acids.

**Table 3:** Gas chromatography assessment of breast adipose tissue fatty acid composition according to the presence of bone metastasis in postmenopausal women.

Fatty Acid *		No Bone Metastasis *n* = 127		Bone Metastasis *n* = 32		Statistics
		Mean	Range	Mean	Range	*p*
**Saturated**						
Myristic acid	14:0	3.00	1.65–5.25	3.04	1.83–4.63	0.76
Palmitic acid	16:0	22.42	16.71–28.13	23.27	18.23–27.58	0.07
Stearic acid	18:0	5.07	1.99–7.82	5.06	2.79–7.66	0.98
	Total SFA	31.36	23.42–40.32	32.25	23.74–37.27	0.22
**Monounsaturated**						
Myristoleic acid	14:1	0.27	0.05–0.51	0.24	0.08–0.48	0.20
Palmitoleic acid	16:1	3.90	1.41–7.49	3.64	1.65–7.97	0.38
Oleic acid	18:1*n-*9c	43.61	36.59–50.57	43.49	38.73–49.16	0.81
Vaccenic acid	18:1*n-*7c	2.03	1.40–3.50	1.99	1.47–3.75	0.67
	Total MUFA	51.14	42.02–60.9	50.63	45.26–58.81	0.51
**Polyunsaturated**						
Linoleic acid	18:2*n-*6c	11.45	6.27–18.76	11.23	5.97–21.49	0.68
Gamma linolenic acid	18:3*n-*6	0.05	0.02–0.10	0.04	0.02–0.16	0.56
Arachidonic acid	20:4*n-*6	0.44	0.20–1.01	0.45	0.23–0.88	0.72
	LC-PUFA *n-*6	1.29	0.55–3.76	1.34	0.71–2.24	0.60
	Total *n-*6	12.82	7.35–20.06	12.64	7.21–22.74	0.75
Alpha linolenic acid	18:3*n-*3	0.60	0.20–1.30	0.60	0.28–1.32	0.96
Eicosapentaenoic acid	20:5*n-*3	0.10	0.02–0.31	0.09	0.03–0.33	0.39
Docosapentaenoic acid	22:5*n-*3	0.30	0.09–0.61	0.43	0.16–0.88	0.17
Docosahexaenoic acid	22:6*n-*3	0.25	0.04–0.54	0.27	0.08–0.87	0.43
	LC-PUFA *n-*3	0.65	0.19–1.20	0.70	0.28–2.08	0.43
	Total *n-*3	1.30	0.69–2.15	1.34	0.65–2.79	0.54
***n-*6/*n-*3 ratio**		10.46	5.49–27.08	9.96	3.42–17.87	0.41

* expressed as % area, SFA: saturated fatty acids, MUFA: monounsaturated fatty acids, PUFA: polyunsaturated fatty acids, LC PUFA: long-chain polyunsaturated fatty acids.

**Table 4:** Demographic and histological characteristics of women with breast cancer with bone metastases (BM) according their menopausal status.

	Premenopausal Women with BM *n* = 21		Postmenopausal Women with BM *n* = 32		Statistics
	Mean or *n* (%)	Range	Mean or *n* (%)	Range	*p*
Age (years)	41.8	33–53	64.4	45–84	<0.0001
BMI (kg/m^2^)	24.8	13–41	25.9	17–40	0.55
Histological size (mm)	36.2	5–70	25.8	5–70	0.05
**Molecular phenotype**					
Luminal A	8 (36.8%)		13 (40.6%)		0.33
Luminal B	4 (21.0%)		12 (37.5%)		
Triple negative	7 (31.6%)		5 (15.6%)		
HER2	2 (10.5%)		2 (6.2%)		
**Other metastatic sites**					
-Visceral	11 (63.1%)		21 (65.6%)		1
-Brain	7 (31.6%)		8 (25.0%)		0.72
Lymphovascular invasion	6 (31.6%)		10 (31.2%)		0.97
Positive axillary LN	12 (63.1%)		16 (50.0%)		1
Multifocality	5 (26.3%)		3 (9.3%)		0.28
Inflammatory Breast cancer	7 (36.8%)		5 (15.6%)		0.20

BMI: body mass index; LN: lymph node; HER2: Human epidermal growth factor receptor 2.
